# Construction and verification of a novel circadian clock related long non-coding RNA model and prediction of treatment for survival prognosis in patients with hepatocellular carcinoma

**DOI:** 10.1186/s12885-023-10508-y

**Published:** 2023-01-17

**Authors:** Zhen Zhang, Wenhui Gao, Xiaoning Tan, Tianhao Deng, Wanshuang Zhou, Huiying Jian, Puhua Zeng

**Affiliations:** 1 Department of Oncology, Affiliated Hospital of Hunan Academy of Traditional Chinese Medicine, 410006 Changsha, P.R. China; 2 School of Chinese Medicine, Hunan University of Chinese Medicine, 410208 Changsha, P.R. China

**Keywords:** Hepatocellular carcinoma, Circadian clock, lncRNA, Immune infiltration, Risk model

## Abstract

Circadian clock genes are significant in the occurrence and development of HCC and long-non coding RNAs (lncRNAs) are closely related to HCC progression. In this study, we aimed to establish a prognostic risk model for HCC. Circadian clock-related lncRNAs expressed in HCC were extracted from The Cancer Genome Atlas. A nomogram was established to predict individual survival rate. Biological processes enriched for risk model transcripts were investigated by Gene Set Enrichment Analysis. Further, we evaluated the relationship between risk score and immune-checkpoint inhibitor-related gene expression level. The Genomics of Drug Sensitivity in Cancer (GDSC) database was used to assess the sensitivity of tumors in high- and low-risk score groups to different drugs. A total of 11 circadian clock-related lncRNAs were included in multi-Cox proportional hazards model analysis to establish a risk model. Univariate and multivariate Cox regression analysis showed that the risk model was an independent risk factor in HCC. The risk model was a significantly associated with the immune signature. Further GDSC analysis indicated that patients in each risk score group may be sensitive to different anti-cancer drugs. QRT-PCR analysis results showed that C012073.1, PRRT3-AS1, TMCC1-AS1, LINC01138, MKLN1-AS, KDM4A-AS1, AL031985.3, POLH-AS1, LINC01224, and AC099850.3 were more highly expressed in Huh-7 and HepG2, compared to LO2, while AC008549.1 were lower expressed. Our work established a prognostic model for HCC. Risk score analysis indicated that the model is significantly associated with modulation tumor immunity and could be used to guide more effective therapeutic strategies in the future.

## Introduction

Hepatocellular carcinoma (HCC) is among the most prevalent malignancies worldwide and the third leading cause of cancer-related death globally. According to GLOBOCAN 2020, there are more than 900,000 new patients with HCC every year, and approximately half of them are Chinese [1]. Due to the absence of obvious symptoms, 80% of patients with HCC have mid to late stages disease at presentation and, therefore, miss the opportunity for surgery [2]. Although radiotherapy and chemotherapy are options for some patients, recurrence and metastases remain significant challenges [3]. As a novel therapeutic method, immune checkpoint inhibitors have exhibited significant benefits in several clinical trials [4]. Nevertheless, due to innate drug resistance and side effects, some patients fail to achieve significant improvement in overall survival (OS) [5–7]. In addition, the 5-year OS rate of HCC is only 20% [8]. Therefore, exploration of new strategies to predict patient survival and improve clinical prognosis is urgently required.

Recently, accumulating evidence from both epidemiological research and preclinical data have shown that circadian clock-related genes are involved in fun regulation of various biological processes and that clock gene dysfunction gives rise to cancer occurrence and development [9, 10]. For example, shift work is a high risk factor of HCC, as evidenced by a strong correlation between long period and of shift work and increased cancer risk [11]. Further, mice lacking circadian clock genes are more prone to tumorigenesis on γ-irradiation than wildtype mice [12]. Moreover, growing evidence supports that disfunction of circadian clock genes is common and may promotes liver carcinogenesis and accelerated cancer progression [11, 13]. The mRNA expression level of circadian clock genes, including *PER1*, *PER2* and *CRY2*, were significantly decreased in HCC tumors relative to adjacent tissue [14], while complete deletion of PER2 disrupts clock-controlled pathways and patterns and promotes the expression of cMYC, resulting in increased susceptibility to development of HCC [13]. Therefore, regulation of the circadian clock has potential be a novel therapeutic strategy for patients with HCC.

Long non-coding RNAs (lncRNA) are a class of regulatory transcripts longer than 200 nucleotides that don’t code protein [15, 16], and the roles of lncRNAs in malignancy occurrence, progression, metabolism, and prognosis have been explored [17]. Recent studies have shown that lncRNAs have vital roles in regulation of circadian clock genes. On the other side, LncRNAs play pivotal roles in regulation of circadian clock genes, having different biological functions in various cancer types [18, 19]. Ming and colleagues showed that a lncRNA highly expressed in HCC can increase the expression of CLOCK to disrupt the circadian rhythm, resulting in the promotion of HCC [20]. In addition, human *LNC-UC* epigenetically modifies transcription of the circadian clock gene, *Rev-erb*α, which restrains inflammation in colitis [21]. However, the prognositic value of circadian clock-related lncRNAs signature in HCC have not been fully determined.

In this study, we aimed to identify circadian clock related lncRNAs differently expressed in HCC and used them to construct a molecular signature model. The results indicate that the developed prognostic circadian clock-related lncRNAs signature is a viable prognostic predictor for HCC. Additionally, correlations between the signature model and clinical factors were explored.

## Materials and methods

### Data collection and pretreatment of datasets and samples

RNA-Seq, lncRNA sequencing data, and related clinical information of HCC were extracted from The Cancer Genome Atlas (TCGA) (https://www.cancer.gov/about-nci/organization/ccg/research/structural-genomics/tcga accessed on 7 June 2022)database. After exclusion of patients from TCGA with incomplete clinical information, 369 HCC samples and 57 normal tissue samples were included in our study. Copy number variation (CNV) data in HCC was download from UCSC Xena (http://genome.ucsc.edu/ accessed on 7 June 2022). A total of 51 circadian clock genes were identified from literatures, including 14 core circadian clock genes and 37 related circadian clock genes [22–25]. A protein–protein interaction (PPI) network was constructed using the STRING (https://cn.string-db.org/) database with a medium confidence setting (0.4). The location of CNV alteration of circadian clock genes on chromosomes were analyzed by R (version 4.1.1) and R Bioconductor package. Circadian clock genes differentially expressed between tumor and normal tissues were determined and visualized using the “limma” package in R. Then, Gene Oncology (GO) and Kyoto Encyclopedia of Genes (KEGG) enrichment analysis for differentially expressed circadian clock genes were performed using “clusterProfiler” package in R.

### Identification of prognostic circadian clock related LncRNAs and construction of prognostic risk assessment model

Pearson correlation analysis was employed to explore correlations between expression of circadian clock genes and expression level of all lncRNA in analyzed samples. threshold values were set as ∣Cor Pearson∣ > 0.4, *P* < 0.001,∣log_2_ fold change (FC)∣ > 1, and false discovery rate (FDR) < 0.05. Correlations between circadian clock- related lncRNAs and clinical characteristics were assessed using the R package “ggpubr”. Univariate Cox regression analysis was applied to filter prognostic circadian clock-related lncRNAs with threshold *P* value < 0.05. The least absolute shrinkage and selection operator (LASSO) regression algorithm was implemented to further screen circadian clock-related lncRNAs strongly associated with the OS, using the “glmnet” R package. Risk scores were calculated using the following formula: Risk score = 

$$\sum \left(PC{1}_{i}\right)+\sum \left(PC{2}_{i}\right)$$

where i represents the prognosis circadian clock-related lncRNAs.

### Assessment of the prognostic risk scores

Patients were randomly divided into training and test group and the risk score for each patient calculated using our scoring model. In all, 220 samples were included in training group and 149 samples were included in test group. According to median risk score, patients were stratified into two groups, high- and low-risk groups. Then, we used Kaplan–Meier survival plot and log-rank test to explore the differences in OS between the two groups. Correlations between risk score and survival status of each patient are illustrated using risk curves and scatter plots.

### Predictive nomogram construction and calibration

Risk scores, sex, tumor grade, and tumor stage were included in univariate and multivariate Cox analyses to determine variables related to prognosis. Then, a nomogram was constructed to evaluate the predictive efficiency of risk scores using “rms” R package. Principal component analysis (PCA) was applied to classify patients according to circadian clock-related lncRNA expression patterns.

### Gene Set Enrichment Analysis (GSEA)

Genes differently expressed between the high- and low-risk groups were identified and GSEA were employed to investigate which biological functions and pathways were enriched for these in two groups. The R package “limma” and “enrichplot” were used for this analysis.

### Relationship between risk score and immune signatures in HCC

The following six methods were used to investigate immune infiltration status: TIMER, CIBERSORT, EPIC, MCPCOUNTER, XCELL, and CIRBER-ABS [26]. Differences immune cell infiltration content between the two groups were investigated using the Wilcoxon signed-rank test. Spearman correlation analysis was employed to explore the correlation between immune infiltration cells and risk score. Heatmap was generated using “ggplot2” in R. Further, the R package “ggstatsplot” was used to evaluate the relationship between risk score and immune-checkpoint inhibitor-related gene expression levels. Immune-related genes were extracted from the ImmPort database (https://www.immport.org), to explore the relationship between risk model and immune chemokines and cytokines.

### Evaluation of predicted sensitivity to therapeutic drugs

Genomics of Drug Sensitivity in Cancer (GDSC), the largest public pharmacogenomics database, was used to assess the predicted sensitivity to different drugs of tumors allocated to the high- and low risk score groups [27]. The prediction process used was the “pRRophetic” where half-maximal inhibitory concentration (IC_50_) was constructed by ridge regression model based on gene expression profiles [28].

### Cell culture and qRT-PCR

Human hepatic cell line (LO2) and HCC cell lines (Huh-7 and HepG2) were obtained from Guangzhou Jennio Biotech Co., Ltd. (Shanghai, China). All cells were maintained in dulbecco's modified eagle medium (Invitro-gen, Carlsbad, CA, USA) and 10% fetal bovine serum (Invitrogen) with 1% penicillin-strepomycin (Invitrogen) at 37%℃ and 5% CO_2_. Based on the manufacturer's protocol, total tissue RNA was extracted using TRIzol regent (Tiangene, China). Subsequently, the RNA was transcribed into cDNA. Then, with the cDNA as the template, qRT-PCR was carried out. Relative mRNA expression was calculated using the 2^−ΔΔCt^ method.

### Statistical analysis

Perl software and R software (Version 4.1.1) with corresponding packages were conducted for statistical analyses and plot drawing. Wilcoxon test were used to compare circadian clock genes between tumor and normal tissues. Immune related signature between high- and low- groups were explored by Wilcoxon test.

## Results

### Landscape of circadian clock genes in HCC

**Fig. 1:**
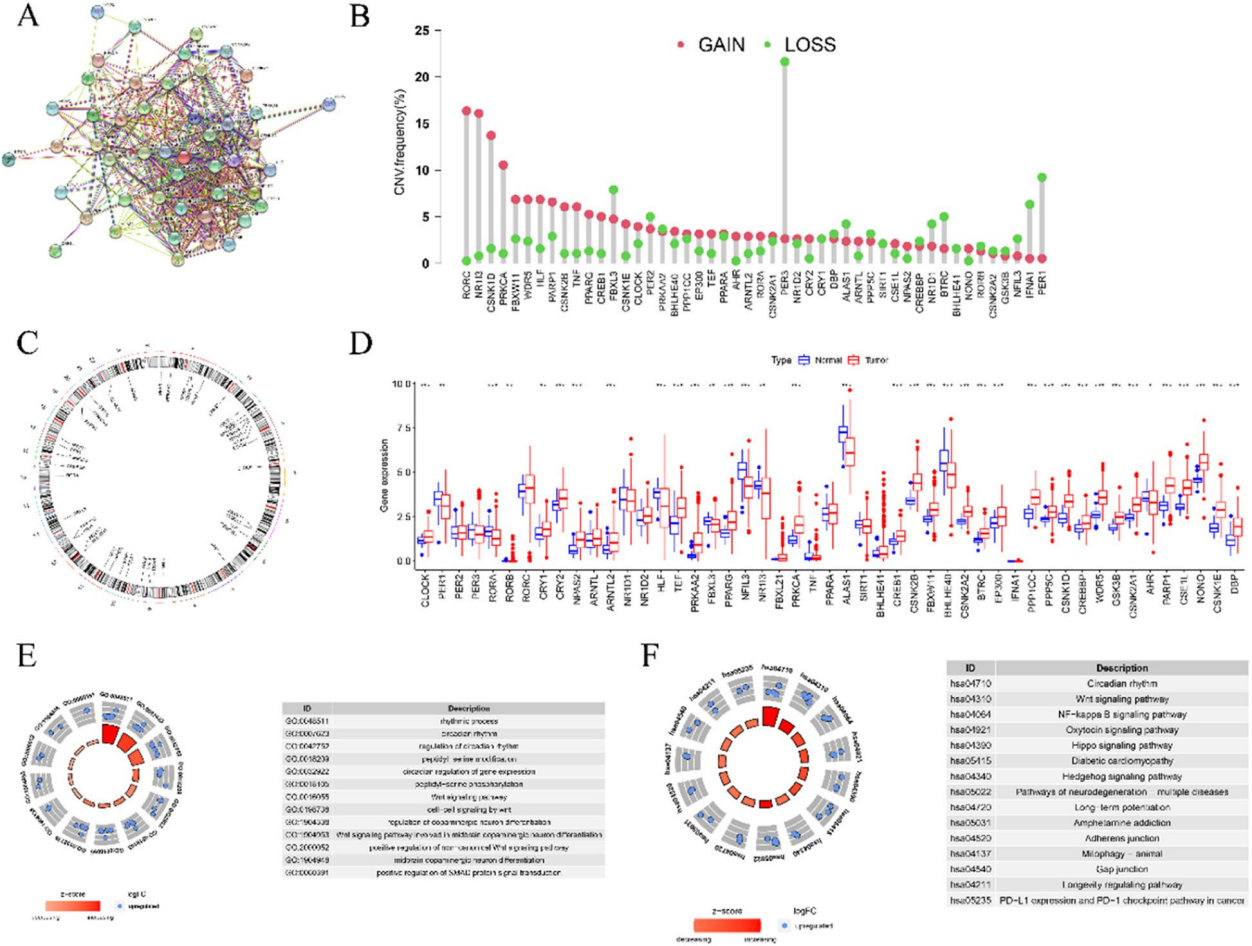
The genetic landscape of circadian clock genes in HCC. **A**. PPI network among circadian clock genes. **B**. the CNV of circadian clock genes. The column represented the alteration frequency. The red points represented amplification of CNV. The green points represent deletion of CNV. **C**. The location of CNV alteration on chromosomes. **D**. The difference of mRNA expression of 51 circadian clock genes between normal and tumor samples. The asterisks represented the statistical *P*-value (^*^P < 0.05; ^**^P < 0.01; ^***^P < 0.001). **E**. GO enrichment of differently expressed circadian clock genes in HCC. F. KEGG enrichment of differently expressed circadian clock genes in HCC

In order to explore the correlation between circadian clock genes, we set medium confidence at 0.4 to analyze PPI. As seen in Fig. 1A, there were 49 nodes and 450 edges and the average node degree was 18.4. In addition, the incidence of copy number variation frequency was summarized in this study. As shown in Fig. 1B, PER3, RORC, and NR1I3 exhibited a widespread frequency of CNV alteration. The location of CNV alteration of circadian clock genes on chromosomes was presented in Fig. 1C. In order to ascertain whether the above genetic variation influence the expression of circadian clock genes patients in HCC, we investigated the mRNA expression of circadian clock genes between normal and cancer tissues in HCC patients. We found that the changes of CNV may be a prominent factor resulting in perturbations on the circadian clock gene expression. The results showed that 37 of 51 genes had a different expression level between normal and cancer tissues in HCC patients (Fig. 1D). Subsequently, we performed GO and KEGG enrichment analysis of these differently expressed genes and found that besides the circadian rhythm, they may play a key role in regulating Wnt signaling pathway, NF-κB signaling pathway, oxytocin signaling pathway, etc. (Fig. 1E-1F).

### Construction of prognosis risk model

**Fig. 2:**
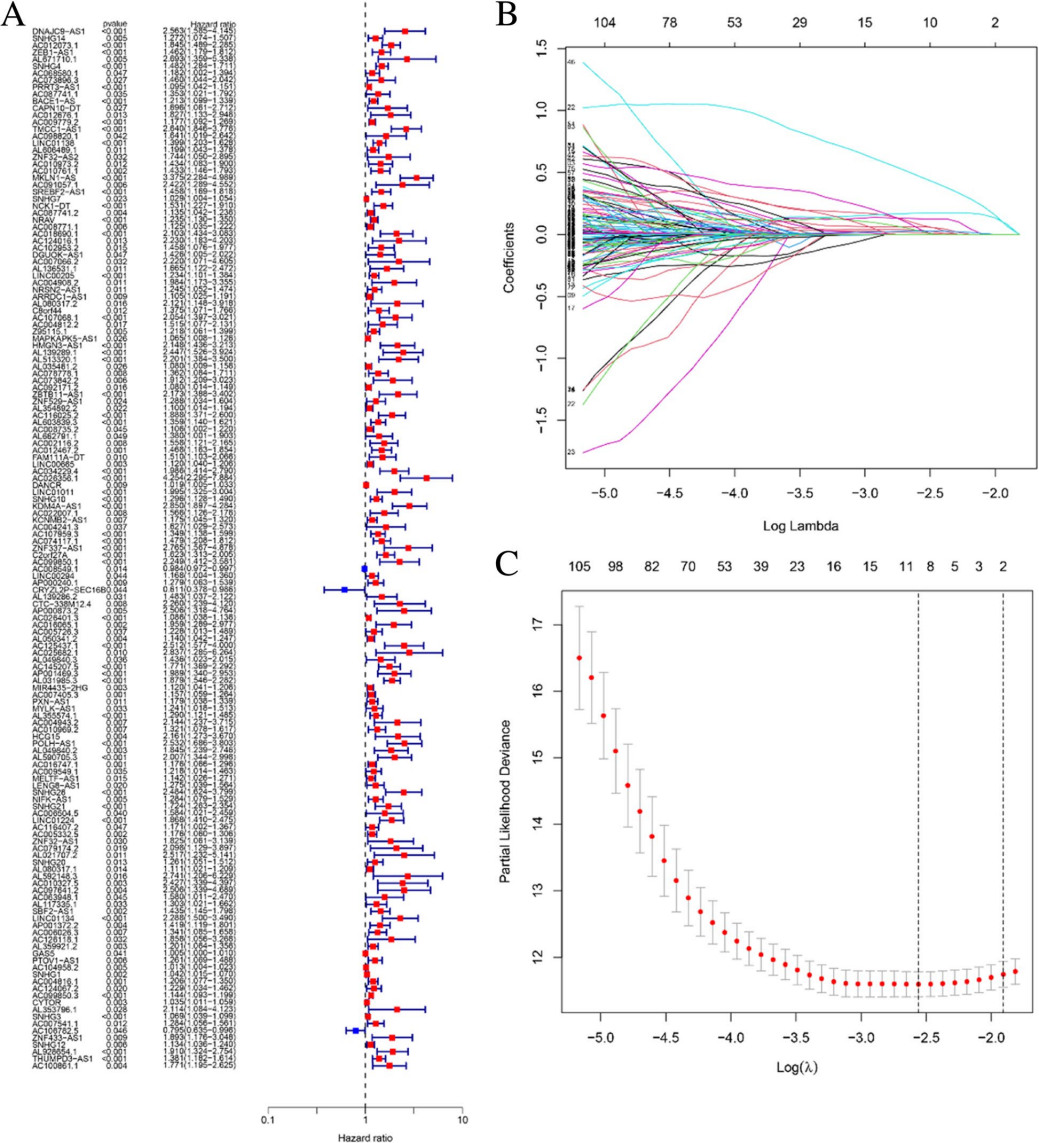
Identification of prognostic circadian clock-related lncRNAs in HCC. **A**. The prognostic lncRNAs extracted by uni-Cox regression analysis. **B**. The tenfold cross-validation for tuning parameter section in the LASSO MODEL. **C**. The LASSO coefficient profile of survival -associated lncRNAs

**Fig. 3:**
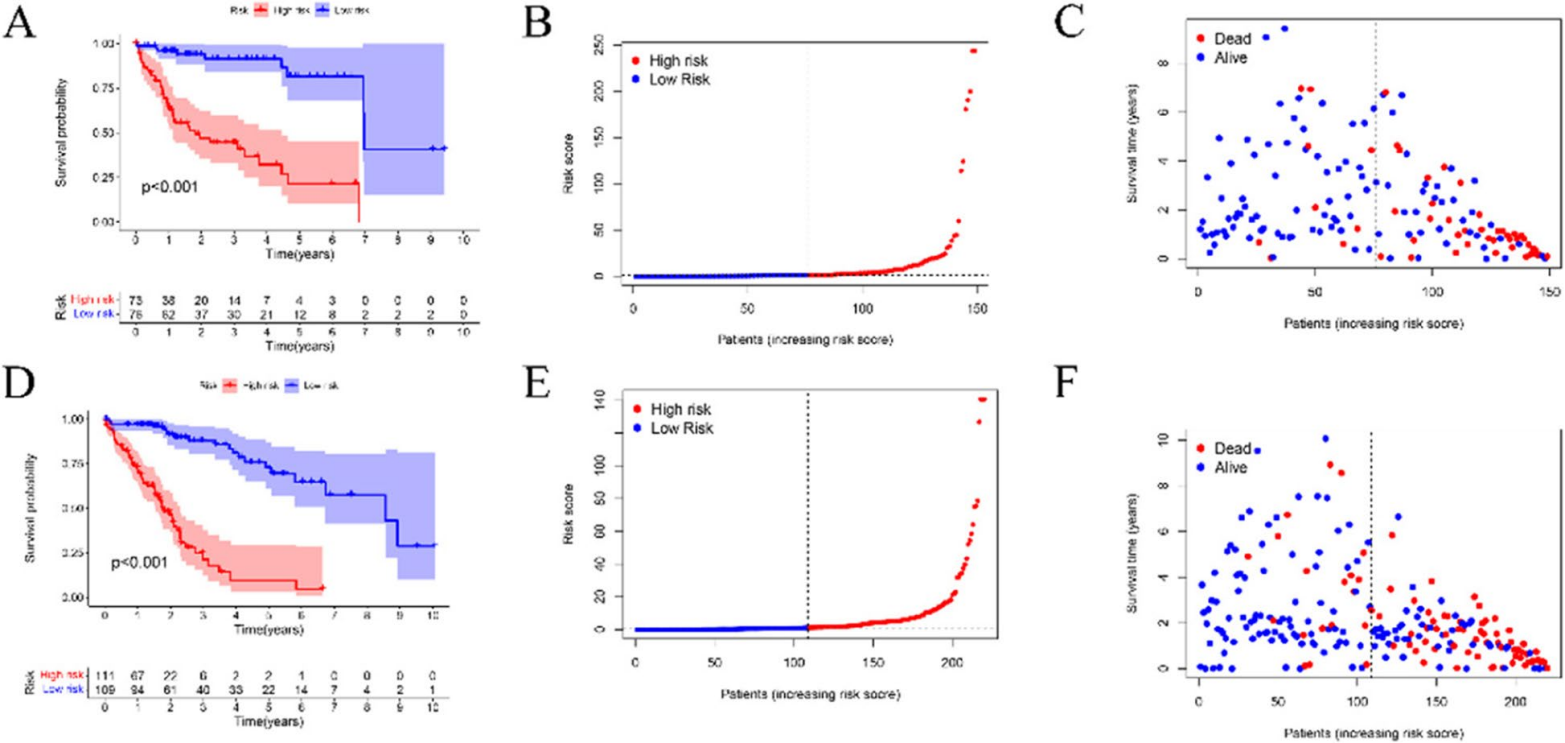
Prognosis of the risk score model in training (**A**-**C**) and test groups (**D**-**F**). A &D. K-M survival curves of OS between two risk group in the training group and test group. B&E. Demonstration of risk model of the training and testing group. C&F. Survival status in training and test groups

**Fig. 4:**
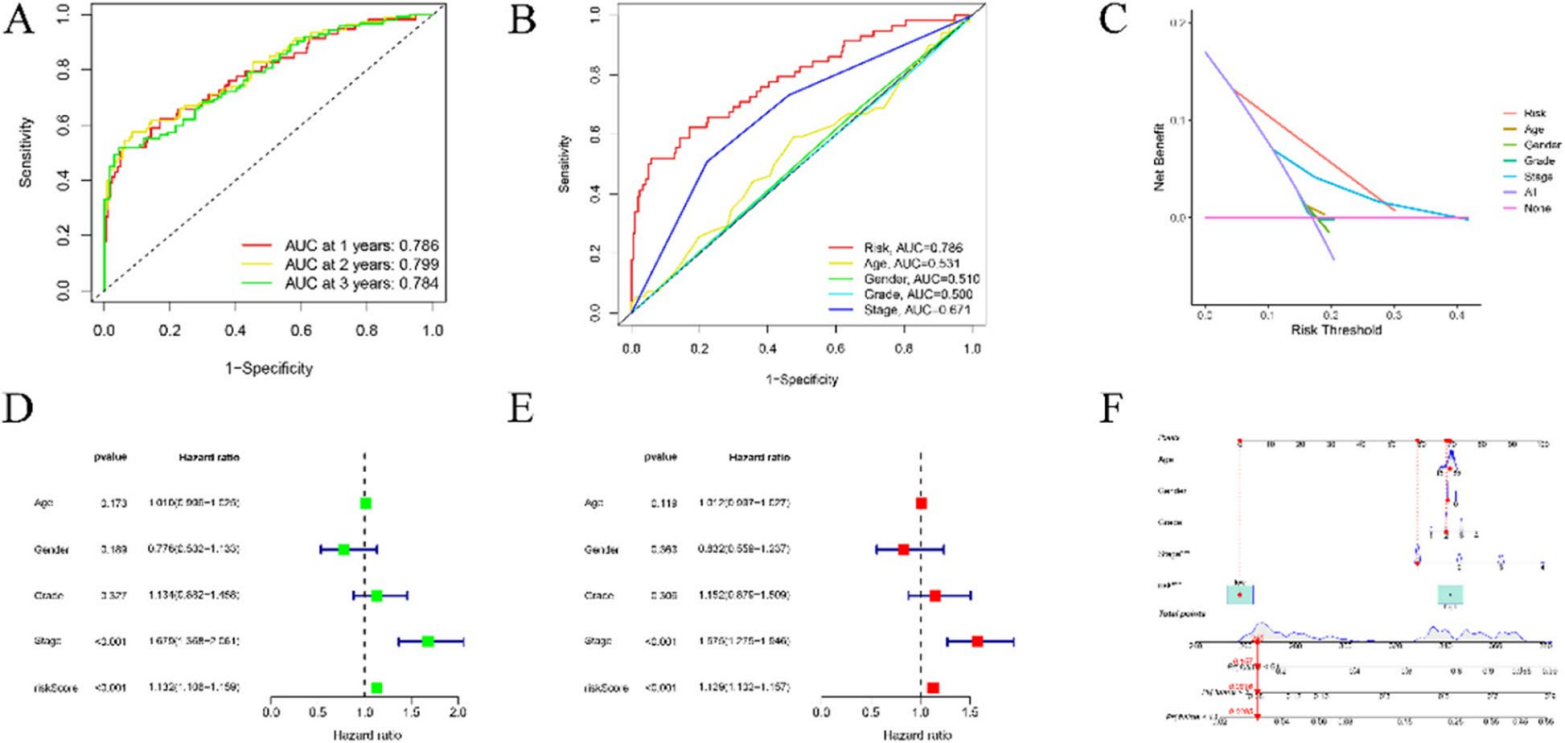
Verification of prognosis risk assessment model. **A**. The 1-, 3-, and 5-year ROC curves of the complete sets. **B**. The ROC curve of risk score and clinic characteristics. **C**. The C-index curves of risk model. **D**. Univariate analyses of clinic characteristic and risk score with OS. **E**. Multivariate analyses of clinic characteristic and risk score with OS. **F**. Nomogram for predicting OS

Firstly, 149 lncRNAs were screened by means of the univariate Cox regression analysis. Then 11 lncRNAs were obtained by LASSO analysis. And 11 (AC012073.1, PRRT3-AS1, TMCC1-AS1, LINC01138, MKLN1-AS, KDM4A-AS1, AC008549.1, AL031985.3, POLH-AS1, LINC01224, AC099850.3) of these lncRNAs were included to establish risk model (Fig. 2A-C). According to training and test groups, the patients set in the high-risk group had a significantly unfavorable prognosis compared to low-risk group (Fig. 3A-F). And in both training and validation group, the AUC was greater than 0.71. The area under the 1-, 3-, and 5-year ROC curve (AUC) was 0.786, 0.799, and 0.784, respectively (Fig. 4A). At the 5-year ROC of the model, the AUC of the risk score was 0.786, indicating strong predictive ability compares with other clinical factors (Fig. 4B-C). The 1-year C-index in the risk model was observed in Fig. 3C. Clinical factors, including age, gender, stage, and grade as well as risk score were included in univariate and multivariate analysis. In univariate and multivariate, the stage and risk score were significantly related to OS (Fig. 4D-E). The hazard ratio (HR) of the risk score was 1.132 and 1.129, respectively. Clinical characteristics of patients with HCC that correlated with Decision Curve Analysis (DCA) parameters were screened. Clinical characters of HCC patients in low and high risk groups were shown in Table 1

**Table 1:** Clinical characters of HCC patients in low and high risk groups

Characteristic	Low risk group	High risk group
n	185	184
Age		
≤ 60 (%)	98(53.0%)	86(46.7%)
> 60 (%)	87(47.0%)	99(53.8%)
Gender		
Male (%)	131(70.8%)	116(63.0%)
Female (%)	54(29.2%)	68(37.0%)
AJCC Stage		
I-II(%)	128(69.2%)	128(69.6%)
III-IV(%)	57(30.8%)	56(30.4%)

### Establishment and nomogram

Based on the risk score and clinical data, a nomogram was established for predicting the 1-, 3-, and 5-year OS incidence. these results indicated that the nomogram may be adapted to precisely access the prognosis of individuals with HCC (Fig. 4F).

### GSEA analysis and immune signature in risk group

**Fig. 5:**
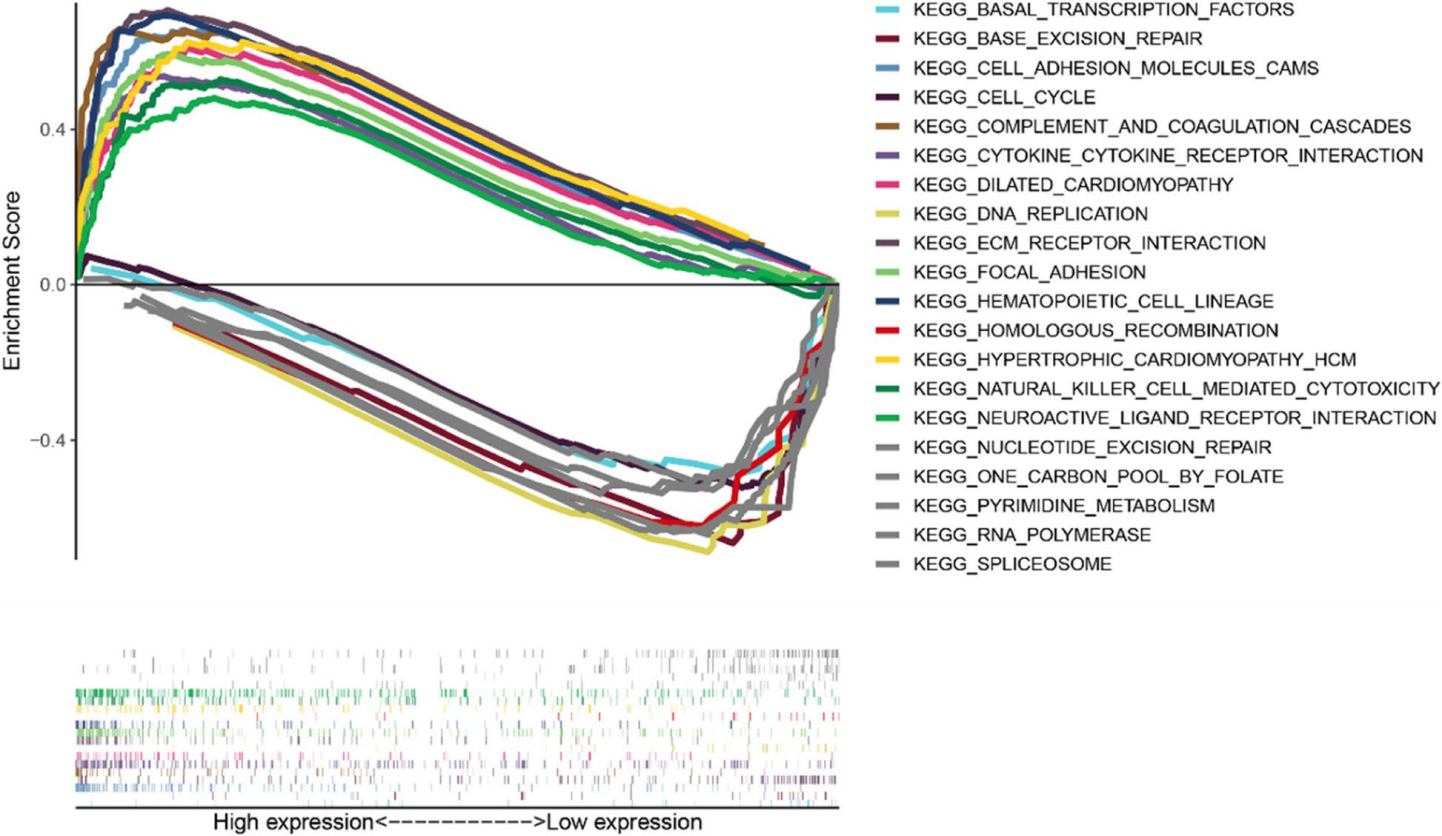
ssGSEA analysis

We conducted KEGG pathway enrichment analysis between different groups. The results showed different biology process between high- and low- group, such as basal transcription factors, base excision repair, cell adhesion molecules cams (Fig. 5).

### Correlation between risk score and immune signature

**Fig. 6:**
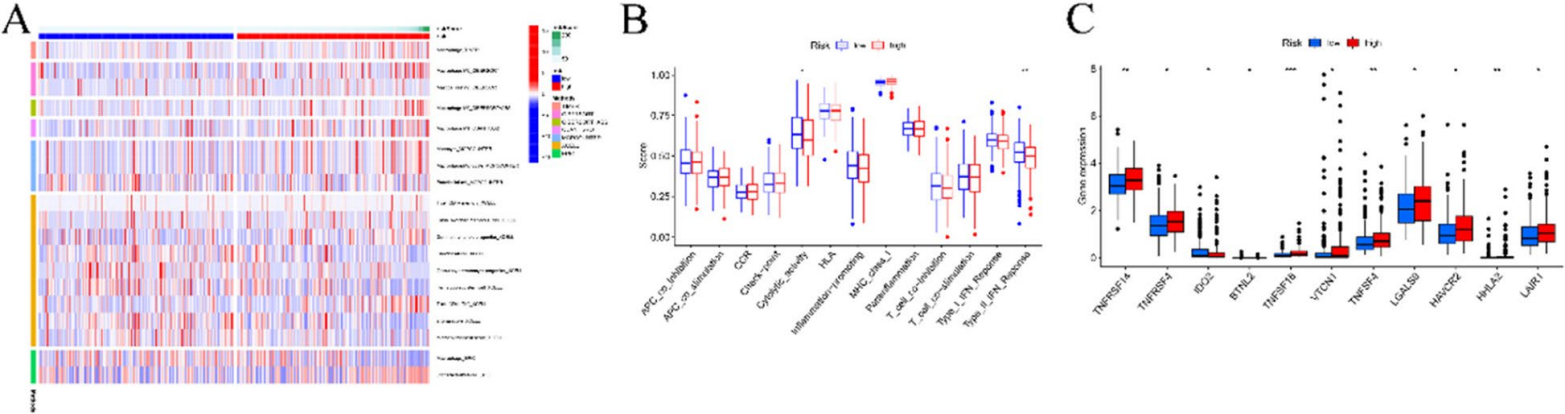
Correlation between risk score and immune signatures. **A**. Correlation between risk score and immune signature. **A**. Heatmap of immune cells in six different databases. **B**. Thirteen immune-related functions. **C**. Expression level of immune-related kinases in two risk model

Based on the previous results, we comprehensively explored the relationship between risk model and tumor immune microenvironment in HCC. As seen in Fig. 6A, the risk score is high related with immune cells, including macrophages, CD4 + T cells, etc. To further explore the difference in immune status between high- and low- groups, we estimated the immune related functional pathways. As can be seen in Fig. 6B, cytolytic activity and type II IFN response were remarkably upregulated in the low-risk group. We further investigated the expression of immune checkpoint molecular and receptors and found that TNFRFSF14, TNFRSF4, IDO2, TNFSF18, VTCN1, TNFSF4, LGALS9, HAVCR2, HHLA2, and LAIR1 gene exhibited significant differences between two risk groups (Fig. 6C). These results indicated that circadian clock related lncRNAs risk model had a significant association with the immune signature.

### Therapeutic drug sensitivity

**Fig. 7:**
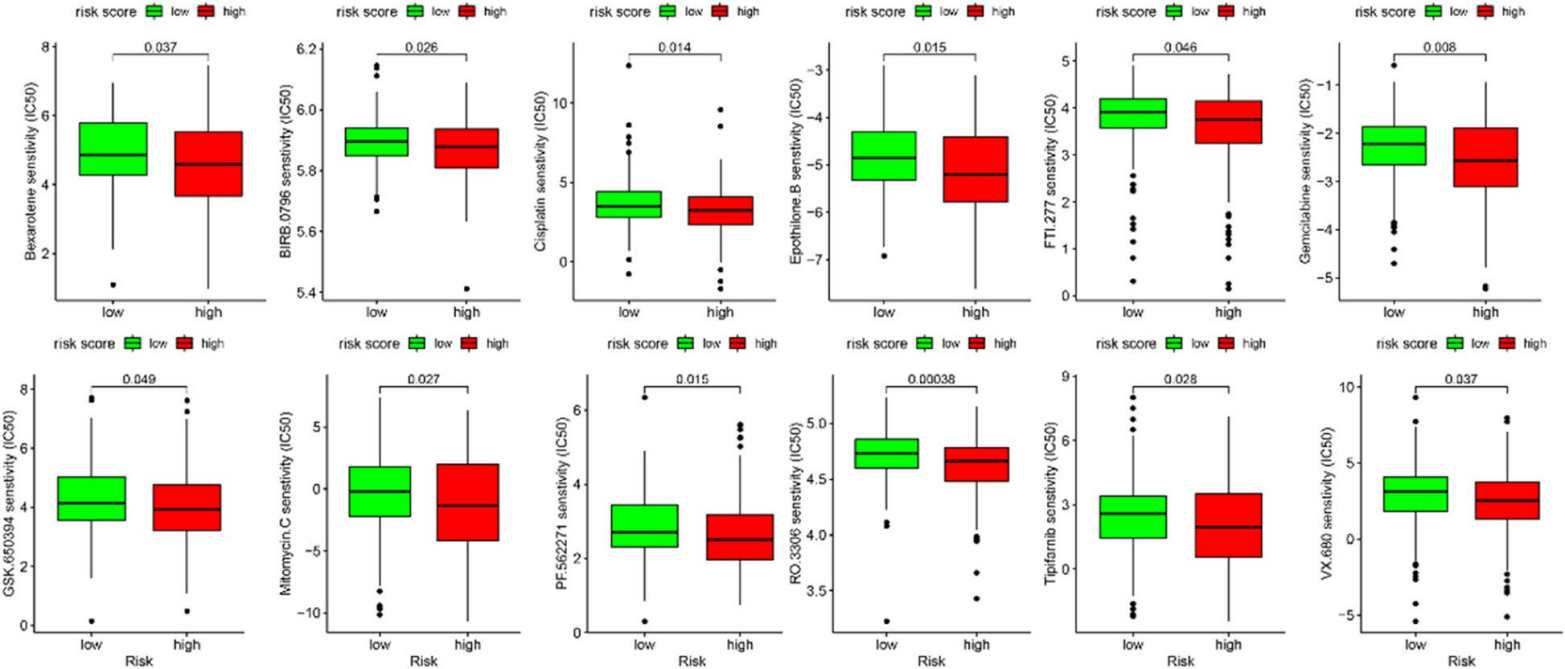
Sensitivity of antitumor drugs with a lower IC50 in high-risk group

**Fig. 8:**
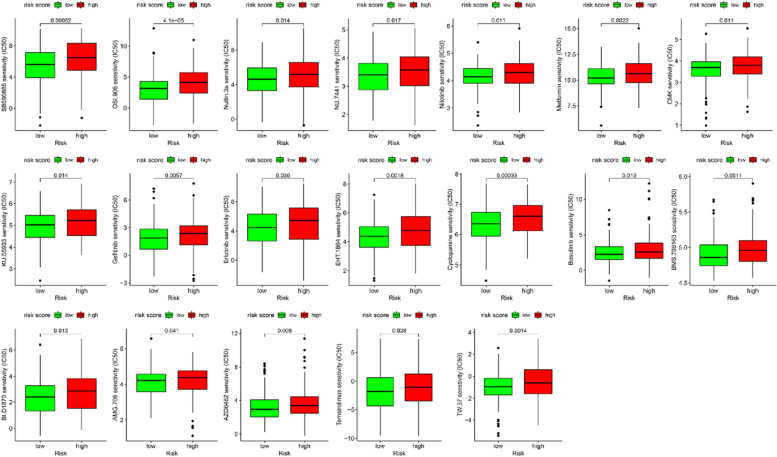
Sensitivity of antitumor drugs with a lower IC_50_ in low-risk group

Considering the diversity of drug treatments in HCC, we further investigated the response of patients with 138 different types of drugs. In details, the GSDC dataset was employed to access the IC50 of the selected drugs based on “pRRophetic” packages. As can be seen in Fig. 7, 15 drugs were identified obviously lower IC50 in high-risk group, while 19 drugs demonstrated significantly lower IC50 in low group (Fig. 8). The finding suggested that risk score model was a potential guider for clinical use of drugs in HCC.

### The expression of 11 circadian clock-related genes in HCC

**Fig. 9:**
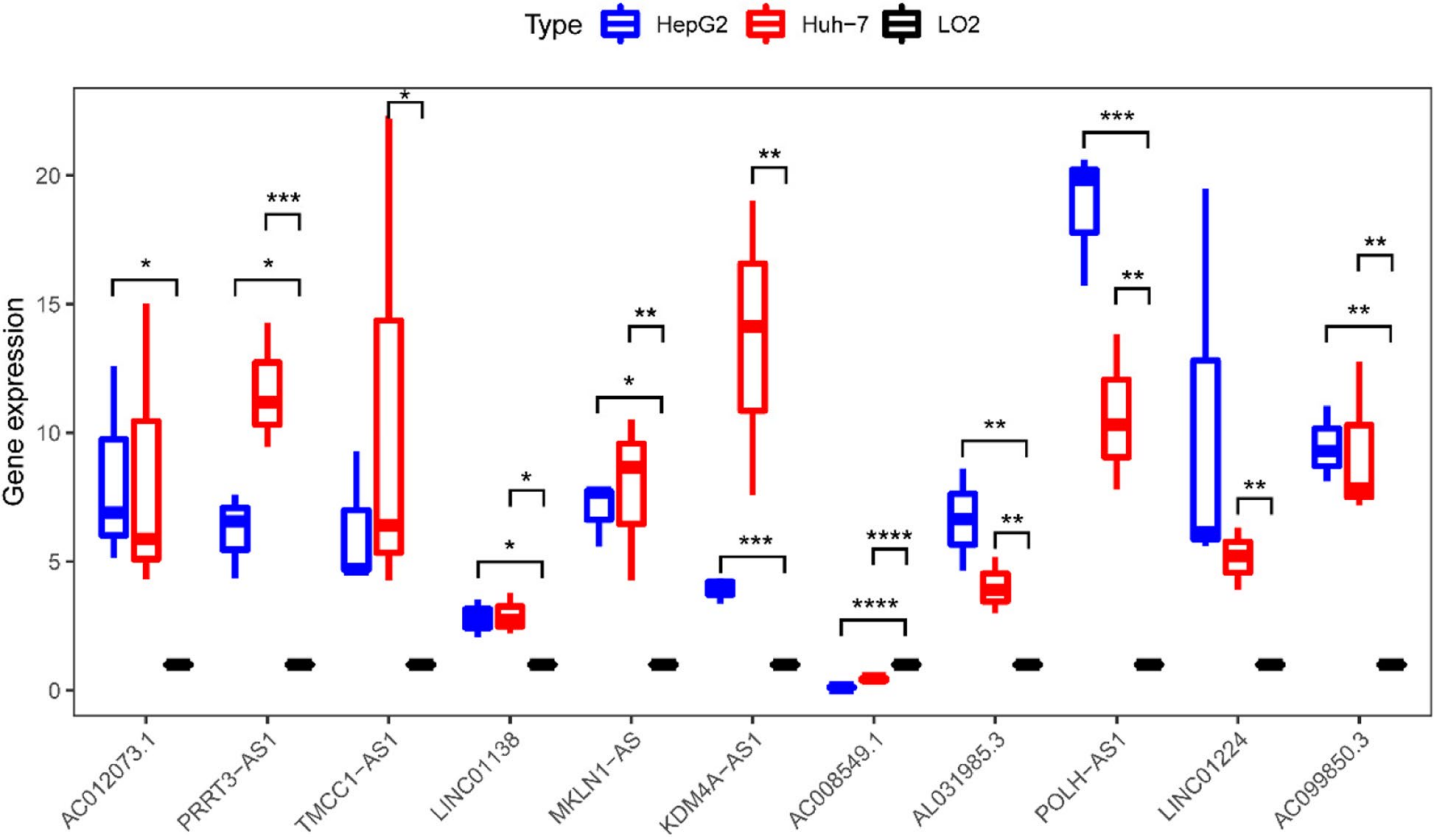
The expression of these 11 lncRNAs in HCC and hepatocyte cell lines

To further explore the expression of these 11 circadian clock-related genes in HCC, LO2 cell line and two HCC cell lines were employed to validate the expression levels of 11 lncRNAs. QRT-PCR analysis results showed that C012073.1, PRRT3-AS1, TMCC1-AS1, LINC01138, MKLN1-AS, KDM4A-AS1, AL031985.3, POLH-AS1, LINC01224, AC099850.3, and AC008549.1 were more highly expressed in Huh-7 and HepG2, compared to LO2, while AC008549.1 were lower expressed. These results indicated that these 11 lncRNAs may play a significant role in HCC (Fig. 9).

## Discussion

In mammals, the signaling pathways which regulate circadian rhythms are universal. Circadian clock genes work together as a whole to regulate the body's life activities. And LncRNAs play pivotal roles in regulation of circadian clock genes, having different biological functions in various cancer types [18, 19]. However, most studies conducted only on a single circadian clock gene, and fewer studies focus on the circadian clock genes as a whole. Recently, prognostic signatures, comprising mRNAs, mRNA expression-based stemness index and microRNAs has been employed to forecast the outcomes of cancers [29, 30].

In this study, we firstly explored the landscape of circadian clock genes. The results indicated that circadian clock genes are tightly linked to each other. Due to the alteration of CNV, 37 of 51 genes had a different expression level between normal and cancer tissues in HCC patients, which was linked to a variety of biological processes, including NF-κB signaling and PD-L1 expression and PD-1 checkpoint pathway in cancer. consistent with the previous study, downregulation of REV-ERBα can significantly enhance the transcription of NF-κB to stimulate the invasion and promote the proliferation of lung adenocarcinoma cell line [31]. Study has shown that as Immune checkpoints, PD-1 or PD-L1 blocked have vastly improved the treatment of various cancers, including HCC [32].

Subsequently, we identified 11 circadian clock-related lncRNAs using LASSO Cox regression analysis. A previous studies established a LASSO prognosis model based on apoptosis-associated genes and showed that the risk model possesses favorable predictive ability [33]. Studies have shown that lncRNAs are correlated with multiple cancers. LncRNA PRRT3-AS1 is able to inhibit the PPARγ gene to promote prostate cancer cell proliferation and inhibit apoptosis and autophagy by activating the mTOR signaling pathway [34]. TMCC1-AS1, AC099850.3 and KDM4A-AS1 were found to be upregulated and promotes cell proliferation and migration [35–37]. MKLN1-AS promotes proliferation and EMT of HCC by mediating the expression of SOX9 [38]. Gong et. identified that LINC01224 are highly expressed in HCC patients with poor prognosis [39]. These results indicated that lncRNAs play significant roles in HCC.

In additional, we also confirmed that the circadian clock-related lncRNAs risk model was an independent risk factor in HCC.

Nomograms is widely employed to predict outcomes of malignant patients [40]. In the current study, a nomogram was established using age, gender, clinic stage, and risk score. The results showed that the model risk and clinic stage were good performance in predicting 1-year, 2-year, and 3-year OS of patients with HCC.

Besides, the results of GSEA indicated different enrichment of genes in KEGG, including basal transcription factors, base excision repair, cell adhesion molecules cams.

The results showed that the risk score is correlated with microphage, mast cell resting, T cell CD4 + memory, etc. Additionally, we further explored the role of risk score and expression of chemokines and cytokines. TNFRFSF14, TNFRSF4, IDO2, TNFSF18, VTCN1, TNFSF4, LGALS9, HAVCR2, HHLA2, and LAIR1 gene exhibited significant differences between two risk groups. Immune function analysis showed that low risk group had a higher leverl of check point and type II IFN response characterized by attenuated antitumor immunity. Overall, the increasement of protumor immunity and immune impairment of antitumor immunity may also be the cause of the poor prognosis.

Further investigation showed that patients with different risk score may be sensitive to different anti-cancer drugs based on GSDMD. These above findings indicated that circadian clock-related lncRNAs risk score was a reliable tool, which was able to use as a predictor for HCC and guide clinical treatment decision to anticancer drugs.

This research also has some limitations. Firstly, our work was performed and validated based on retrospective data. Secondly, the correlation between risk score and immune infiltration needs further experiments to validate.

## Conclusion

In conclusion, our work established a circadian clock-related lncRNAs prognostic model for HCC, which is an independent factor related to OS. Besides, this work indicated that risk score is a significant role in modulating tumor immunity and guide more effective therapeutic strategies in the future.

## Data Availability

The data presented in this study can be found in online repositories (https://www.cancer.gov/about-nci/organization/ccg/research/structural-genomics/tcga).
